# The Hsc70 system maintains the synaptic SNARE protein SNAP-25 in an assembly-competent state and delays its aggregation

**DOI:** 10.1016/j.jbc.2024.108001

**Published:** 2024-11-16

**Authors:** Karishma Bhasne, Antonia Bogoian-Mullen, Eugenia M. Clerico, Lila M. Gierasch

**Affiliations:** 1 Department of Biochemistry & Molecular Biology, University of Massachusetts, Amherst Massachusetts, USA; 2 Department of Chemistry, University of Massachusetts, Amherst Massachusetts, USA

**Keywords:** Hsp70, CSPα, chaperone, neurotransmission, SNAP-25, SNARE complex, protein aggregation

## Abstract

The complex mechanism of synaptic vesicle fusion with the plasma membrane for neurotransmitter release is initiated by the formation of the SNARE complex at the presynaptic terminal of the neuron. The SNARE complex is composed of four helices contributed by three proteins: one from syntaxin (localized at the plasma membrane), one from synaptobrevin (localized at the synaptic vesicle), and two from the intrinsically disordered and aggregation-prone synaptosomal-associated 25 kDa protein (SNAP-25), which is localized to the plasma membrane by virtue of palmitoylation of cysteine residues. The fusion process is tightly regulated and requires the constitutively expressed Hsp70 chaperone (Hsc70) and its J-protein co-chaperone CSPα. We hypothesize that Hsc70 and CSPα cooperate to chaperone SNAP-25, disfavoring its aggregation and keeping it in a folding state competent for SNARE complex formation. To test this hypothesis, we used a bottom-up approach and studied the interaction between Hsc70 and CSPα with SNAP-25 *in vitro*. We showed that the aggregation of SNAP-25 is delayed in the presence of Hsc70 and CSPα. Using a peptide array that spans the sequence of SNAP-25, we identified three potential Hsc70-interacting sequences and designed peptides containing these sequences to test binding in solution. We characterized the interaction of SNAP-25-derived peptides with Hsc70 and CSPα using a combination of biochemical and biophysical techniques, including native-PAGE, binding affinity by fluorescence anisotropy, ATPase-activity of Hsc70, and NMR. We have identified an Hsc70 binding site within SNAP-25 that is likely to represent the site used in the cell to facilitate SNARE complex formation.

Effective communication between neurons occurs *via* the release of neurotransmitters at the synaptic junction (1, 2, 3). Delivery of synaptic vesicle contents to the synaptic cleft relies on vesicle fusion with the plasma membrane at the presynaptic terminal of the neuron (4, 5). This process requires the formation of a four-helical bundle known as the SNARE complex (Fig. 1*A*), where proteins from the vesicle (v-SNARE) and the target plasma membrane (t-SNARE) interact *via* their 60-residue-long SNARE motifs to dock the vesicle at the membrane (6, 7). The SNARE complex is formed by one helix from synaptobrevin (also known as VAMP2) at the vesicle and three helices from the target plasma membrane proteins, two from the synaptosomal-associated 25 kDa protein (SNAP-25), and one from syntaxin-1. This four-helix bundle is stabilized by hydrophobic residues in all helices, with central polar residues that lead to classification of the SNARE complexes as R- and Q-SNAREs (8, 9).

**Figure 1:**
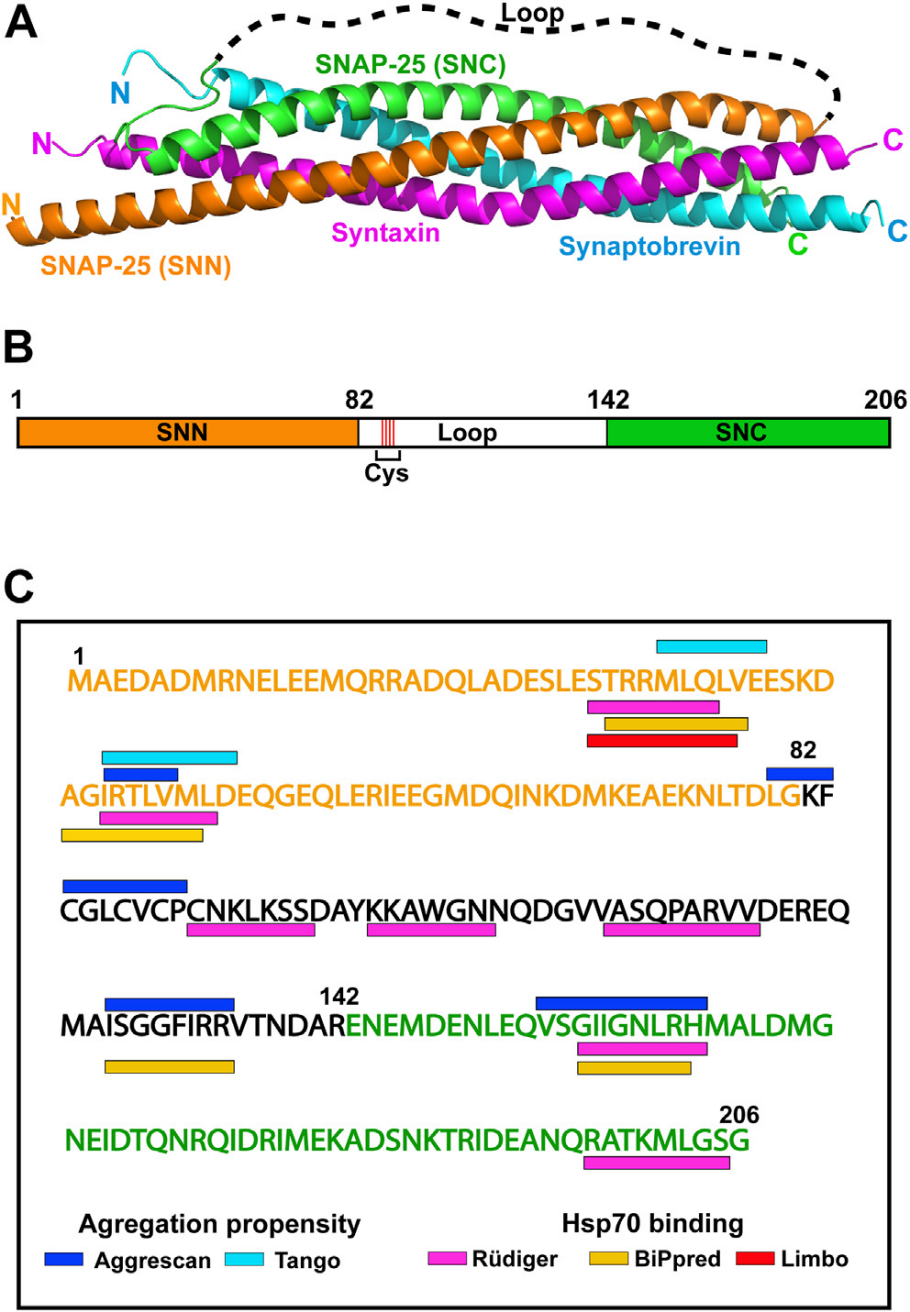
**The SNARE complex and SNAP-25.***A*, crystal structure of the SNARE complex (PDB 1sfc) depicting four helices from syntaxin (*pink*), synaptobrevin (*blue*), and SNAP-25 (SNN domain (*orange*), SNC domain (*green*), and loop region (*black*)). *B*, schematic representation of SNAP-25 showing the N-terminal SNN (*orange*), loop (*black*), and C-terminal SNC (*green*) regions. *C*, amino acid sequence of SNAP-25 showing the SNN (*orange*), loop (*black*), and SNC (*green*) regions, aggregation-prone sites predicted using AGGRESCAN (*blue*) and TANGO (*cyan*), and Hsc70-binding sites predicted using Limbo (*orange*) (31), BiPPred (*yellow*) (32), and Rϋdiger (*pink*) (33). PDB, Protein Data Bank.

Thus, SNAP-25 is a key player in synaptic vesicle fusion. Our understanding of the mechanisms guiding the incorporation of SNAP-25 into the SNARE complex remains limited. Previous studies found that the genetic polymorphism of SNAP-25 was correlated with Alzheimer’s disease (AD) and Parkinson’s disease (PD) progression. In both AD and PD patients, the modulation of SNAP-25 levels in the cortex is associated with neuronal degeneration (10, 11, 12). SNAP-25 is aggregation-prone, intrinsically disordered, and negatively charged (13, 14). It consists of two domains, the 82-amino acid long N-terminal domain (SNN or Sn1) and the 95-amino acid long C-terminal domain (SNC or Sn2) (Fig. 1*B*). Both SNN and SNC form coiled coils in the SNARE complex. A 29-amino acid linker containing four Cys residues connects SNN and SNC (7, 15). These Cys residues are modified in the cell by palmitoylation and acylation within 20 min of SNAP-25 biosynthesis, and this region of SNAP-25 mediates its anchoring to the membrane (16).

The ability of SNAP-25 to remain in a state competent for assembly into the SNARE complex is essential for synaptic vesicle fusion (17, 18), and yet it presents several fundamental questions: How is its aggregation inhibited? How is its tendency to fold modulated so it occurs cooperatively with the other SNARE proteins to form the SNARE complex at the right time and place? While synaptobrevin and syntaxin have known chaperoning factors that support their productive incorporation into the SNARE complex: Munc18 and Munc13, respectively (19, 20, 21), little is known about the cellular factors that support successful SNAP-25 addition to the complex.

Previous studies showed that SNAP-25 interacts with the heat shock protein, heat shock cognate 70 (Hsc70), which has led to the hypothesis that the interaction with this chaperone supports the ability of SNAP-25 to participate productively in SNARE complex assembly (22, 23). In addition, a specialized class C heat shock protein-40 (Hsp40 or J-protein) co-chaperone, the cysteine-string protein-α (CSPα), resides on the synaptic vesicle and is proposed to work with Hsc70 to facilitate SNARE complex formation (14, 23, 24, 25). A small glutamine-rich tetratricopeptide repeat protein, SGT, has been implicated in the Hsc70 system action at the synaptic vesicle as well, but there are other data questioning its role (26, 27). The importance of the Hsc70 system in vesicle fusion is supported by the observation that mutations in CSPα lead to neurodegeneration in humans, which is mimicked in CSPα-KO mice and flies (14, 28).

How the Hsc70/CSPα system works with SNAP-25 to support productive SNARE complex formation has not been explored previously. We have tested the hypothesis that Hsc70 and CSPα maintain SNAP-25 in a conformation compatible with SNARE complex formation, thus facilitating membrane fusion for exocytosis. Importantly, we found that the Hsc70 system interacts with SNAP-25 and delays its aggregation *in vitro*. We characterized the interaction between SNAP-25 and Hsc70/CSPα using a combination of biochemical and biophysical techniques. We identified three distinct Hsc70-binding sites on SNAP-25 and proposed a model in which binding of Hsc70 to one of these sites modulates SNAP-25 assembly into the SNARE complex.

## Results

### Hsc70 delays SNAP-25 aggregation

Outside of the SNARE complex, SNAP-25 is metastable and tends to self-associate and aggregate *in vitro* (22). SNAP-25 aggregates may also form *in vivo*, as they were observed in the brain tissues of schizophrenia patients (13). In order to identify the aggregation-prone sequences in SNAP-25, we used TANGO (29) and AGGRESCAN algorithms (30) and predicted five sites to be aggregation prone (Fig. 1*C*). Based on previous pull-down studies showing that the constitutively expressed chaperone Hsc70 interacted with SNAP-25, we hypothesized that Hsc70 inhibits aggregation and keeps SNAP-25 in the folding competent state.

We searched for possible 70-kDa heat shock protein (Hsp70) binding sites in the sequence of SNAP-25 using published algorithms (Limbo (31), BiPPred (32), and Rudiger (33)) and identified eight different regions (Fig. 1*C*). In many cases, the aggregation-prone sites on SNAP-25 predicted by AGGRESCAN and TANGO overlap or are juxtaposed with the predicted Hsp70 binding sites, supporting the hypothesis that Hsc70 keeps SNAP-25 in an unfolded state and disfavors its aggregation.

SNAP-25 has been shown by CD to be disordered with nascent helical tendencies (34) (Fig. S1). SNAP-25 has four native cysteines that are palmitoylated upon interaction with the membrane. For our studies we used SNAP-25 CS mutants where the native Cys are mutated to Ser. The SNAP-25 CS mutant was used in a previous report (35), and we observed that the CD signature of SNAP-25 is conserved in the mutant (Fig. S1). For all of our studies, the SNAP-25 CS mutant was used unless otherwise mentioned. We assessed formation of the Hsc70-SNAP-25 complex by CD and found no significant conformational change in SNAP-25 and Hsc70 upon interaction (Fig. S1).

**Figure 2:**
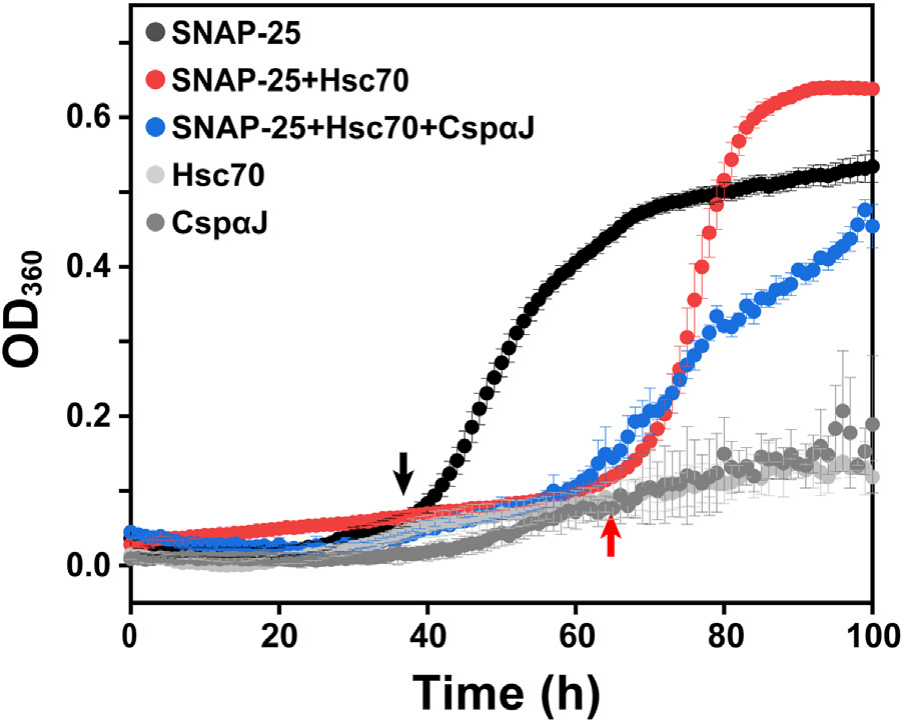
**Hsc70 delays the aggregation of SNAP-25.** Aggregation kinetics of SNAP-25 (50 μM) at 37 °C. Optical density of SNAP-25 was recorded at 360 nm in the absence (*black*) and in the presence of Hsc70 (5 μM, *red*), and Hsc70+CSPαJ (5 μM each, *blue*). Hsc70 (*gray*) and CSPαJ (*dark gray*) alone exhibit no aggregation.

To test our hypothesis that Hsc70 inhibits SNAP-25 aggregation, we monitored the aggregation kinetics of recombinantly expressed SNAP-25 in the absence and presence of Hsc70 by following optical density at 360 nm (Fig. 2). The time course of SNAP-25 aggregation exhibited typical nucleation-dependent kinetics with a lag phase of ∼38 h. This lag time was elongated to ∼65 h in the presence of Hsc70. In neurons, Hsc70 works in collaboration with its co-chaperone CSPα. Here, we show that Hsc70 and CSPα also cooperate *in vitro* to abolish the aggregation of SNAP-25. Figure 2 shows that the presence of CSPαJ (an 82-amino acid fragment of CSPα containing the J-domain) elongates the lag time of SNAP-25 aggregation caused by Hsc70. Estimating the lag time proved challenging as the reaction did not reach saturation even after 100 h, suggesting collaborative efforts between Hsc70 and CSPαJ in prolonging SNAP-25 aggregation. SNAP-25 aggregation kinetics are further elongated by the presence of nucleotide exchange factor Apg2 with Hsc70-CSPαJ. (Fig. S2*A*). Apg2 is a member of the Hsp110 family, which is known to suppress protein aggregation (36). Therefore, we tested the effect of Apg2 alone on SNAP-25 aggregation and observed no significant effect. (Fig. S2*B*).

### Hsc70 interacts with both the SNN and the SNC domains of SNAP-25

**Figure 3:**
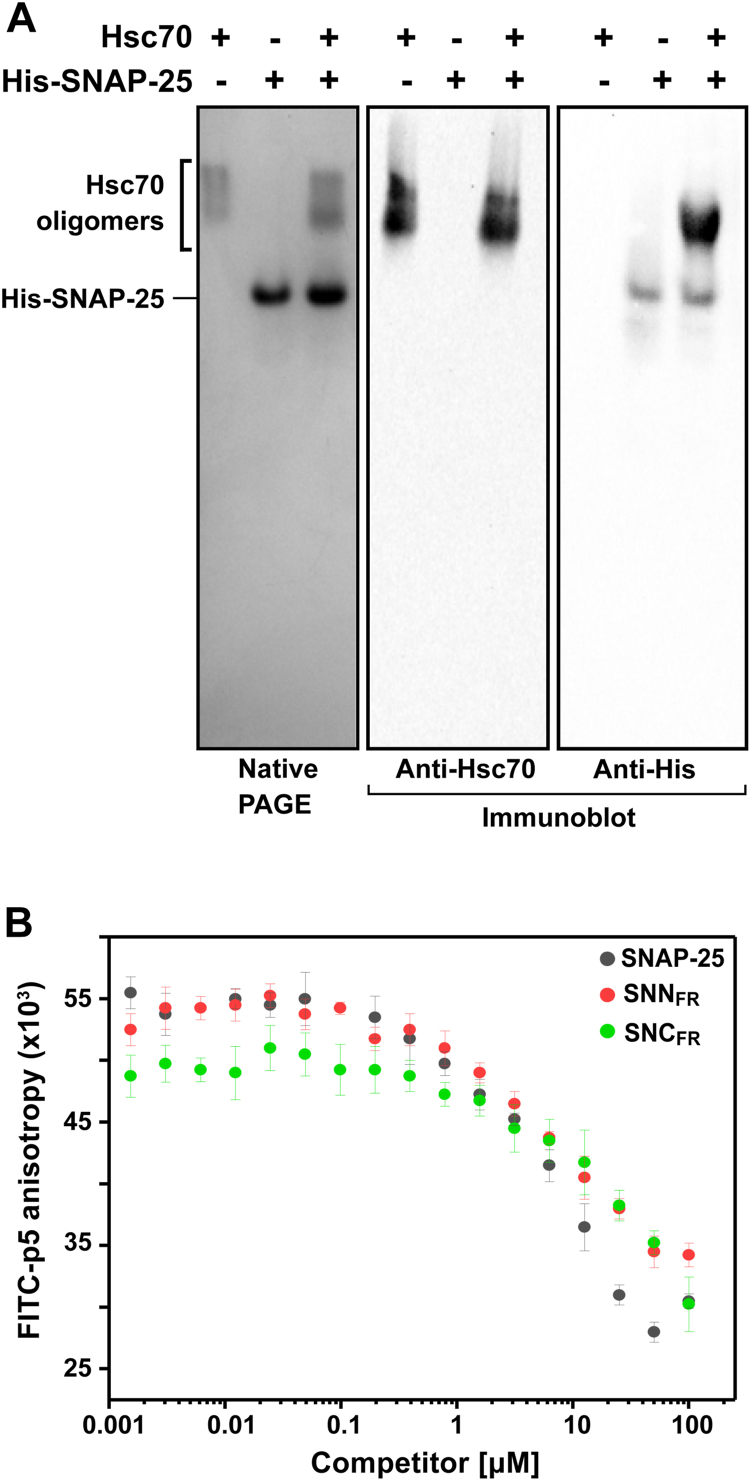
**Hsc70 forms complexes with SNAP-25.***A*, native PAGE of SNAP-25 (10 μM) in the presence and absence of Hsc70 (2 μM). The difference in signal intensity of His-SNAP25 probed with antibody is likely due to altered epitope exposure upon formation of the SNAP-25-Hsc70 complex. *B*, binding of SNAP-25 (*black*), SNAP-25 SNN (SNN_FR_, *orange*), and SNC (SNC_FR_, *green*)) fragments to Hsc70 by competition followed by fluorescence anisotropy. Increasing concentrations of the competitors were added to a complex of Hsc70 (5 μM) and FITC-p5 peptide (50 nM). Error bars represent SDs from three independent experiments.

We initially repeated the previously published pull-down assay (22) to confirm that in our hands we could demonstrate an interaction of His-tagged SNAP-25 and Hsc70. As shown in Figure 3*A*, a complex can be seen in native PAGE of Hsc70 and SNAP-25, using Western blot detection by anti-Hsc70 and anti-His antibodies.

To pin down the regions in SNAP-25 where Hsc70 binds we expressed the individual SNN and SNC fragments of SNAP-25 and tested their ability to compete for Hsc70 binding to an FITC-labeled p5 peptide (ALLLSAPRR). As shown in Figure 3*B*, both the SNN and SNC fragments and full-length SNAP-25 competed with p5 peptide for binding to Hsc70.

### Hsc70 binds to SNAP-25 at three major sites

**Figure 4:**
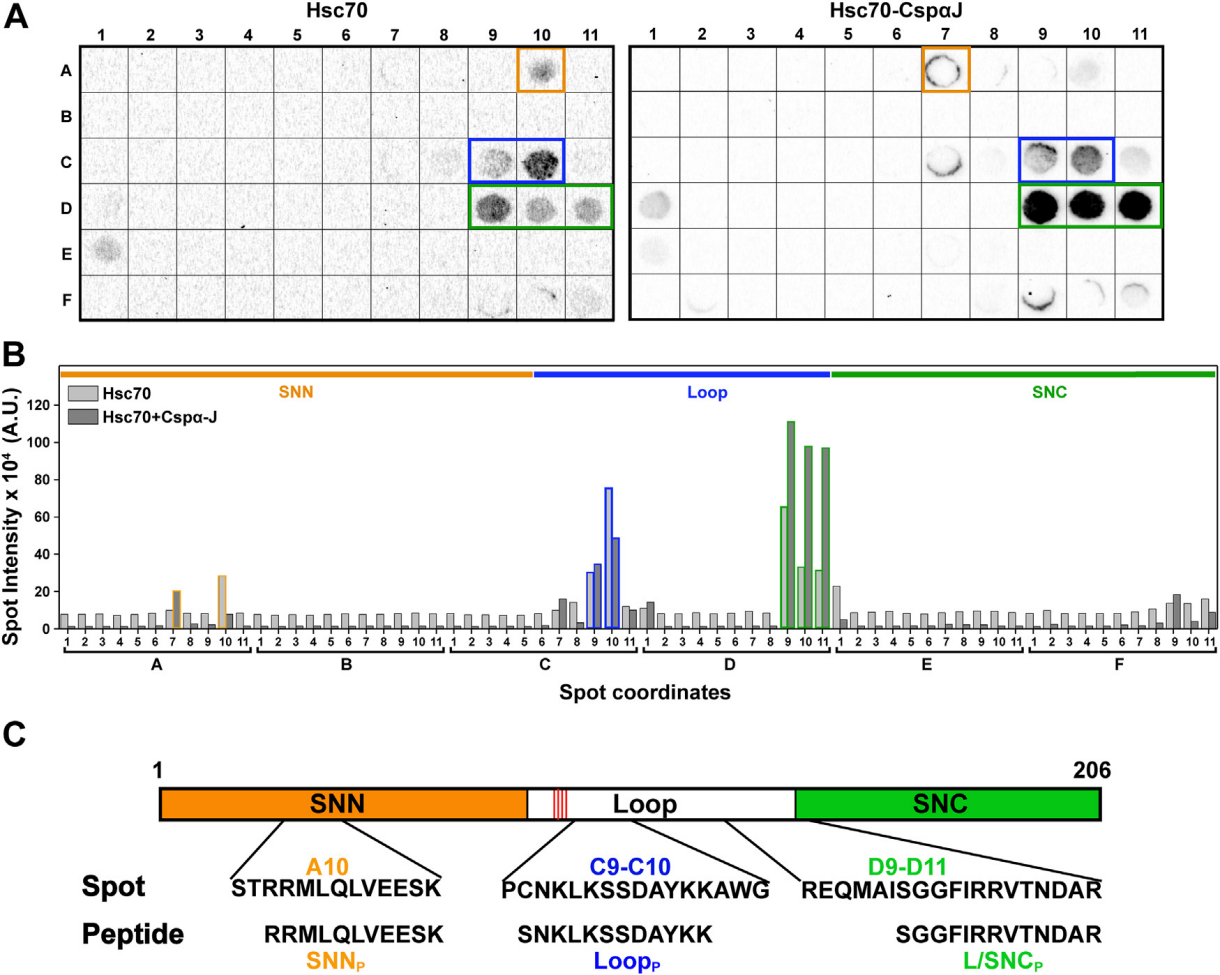
**SNAP-25 peptide arrays show three major Hsc70 binding sites.***A*, cellulose-bound peptide arrays spanning the sequence of SNAP-25 were screened for Hsc70 (*left*) and Hsc70-CSPαJ (*right*) binding. *B*, quantification of the spots from Figure 4*A* using ImageJ (Hsc70 (*gray*) and Hsc70-CSPαJ (*dark gray*)). Signals corresponding to SNN, Loop and Loop-SNC peptides are marked in *orange*, *blue*, and *green*, respectively. *Orange*, *blue*, and *green lines* at the *top* of the graph indicate the SNN, Loop and SNC regions, respectively. *C*, schematic representation of SNAP-25 showing the Hsc70 binding sequences (SNN (*orange*), Loop (*black*), and Loop-SNC (*green*)) identified in the peptide array.

To explore in greater depth the binding sites for Hsc70 in SNAP-25, we screened a cellulose-bound peptide array spanning the full SNAP-25 sequence with 13-residue long peptides, overlapping by ten residues, and probed with Hsc70 in the presence of a two-fold excess of ATP and ten-fold excess of ADP. The binding of Hsc70 to sites in the array was then visualized with an anti-Hsc70 primary antibody and a horse radish peroxidase-tagged secondary antibody to enable visualization by chemiluminescence (Fig. 4*A*). To quantitatively analyze the array, we used ImageJ (https://imagej.net/ij/) and plotted the intensity under the area for each spot. Three major Hsc70 binding sites were identified on SNAP-25. One site is located in the SNN, one in the LOOP, and one in the LOOP-SNC region (called SNC site) (Fig. 4*A*) absorb (Fig. 1*C*). The results show that there are three strong Hsc70 binding sites on SNAP-25 along with some minor binding sites. The binding site in the LOOP region is very close to the cysteine residues that become palmitoylated as SNAP-25 moves to its membrane-localized state. It is formally possible that Hsc70 binds to SNAP-25 in the initial un-palmitoylated state and dissociates upon palmitoylation and association with the plasma membrane. However, it can be assumed that the binding of this region to Hsc70 is not implicated in the events directly preceding SNARE complex assembly, as it will not be accessible to the chaperone. Therefore, we posit that chaperone binding to the SNN and SNC regions plays a mechanistic role in facilitating productive SNAP-25 association with the other SNARE proteins.

We tested the effect of CSPα on Hsc70 binding to SNAP-25 by probing the binding of Hsc70 and CSPαJ to the SNAP-25 peptide array (Fig. 4, *A* and *B*). Figure 4*B* shows that in the presence of CSPαJ, Hsc70 binds to similar sites in SNAP-25 as it did in its absence (Fig. 4, *A* and *B*) indicating that the presence of the co-chaperone does not change the specificity of Hsc70 binding to SNAP-25.

### SNN and SNC peptides interact with the canonical binding site of Hsc70 in solution

**Figure 5:**
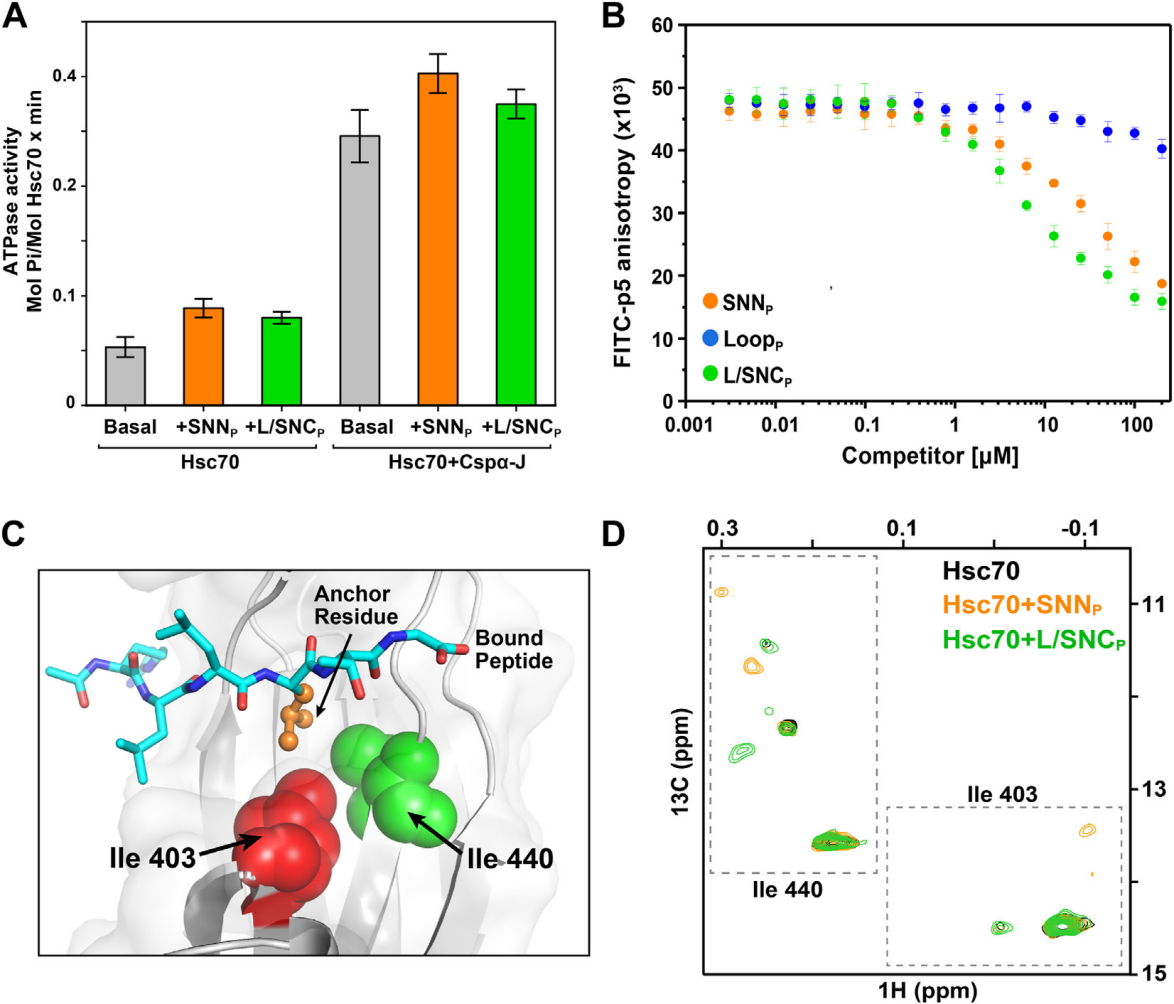
**SNN and SNC peptides interact with the canonical substrate-binding site of Hsc70.***A*, ATPase activity of Hsc70 (2 μM) in the absence (*gray*) and presence of 200 μM SNN_P_ (*orange*) and SNC_P_ (*green*) peptides. *Error bars* represent the SDs from three independent experiments. *B*, binding of SNAP-25 (*black*), SNN_P_ (*orange*), Loop_P_ (*blue*), and SNC_P_ (*green*) peptides to Hsc70 followed by competition for FITC-p5 peptide using fluorescence anisotropy. Increasing concentrations of the competitors were added to a complex of Hsc70 (5 μM) and FITC-p5 peptide (50 nM). Error bars represent SDs from three independent experiments. *C*, structure of the canonical substrate-binding site of human HspA1 (PDB 4po2) illustrating the location of Hsc70 residues Ile 403 and Ile 440 in *red* and *green spheres*, respectively (N.B., Hsc70 and HspA1 share ∼60% sequence identity). The SBD is shown in *gray*, bound substrate in *cyan sticks*, and the side chain of the residue occupying the 0^th^ position in the chaperone (“anchor residue”) in *orange ball* and *sticks*. For better visualization of the substrate-binding site, residues 512 to 613, 405 to 408, and 431 were removed in this illustration of the structure. *D*, the Ile region of the ^1^H-^13^ C HMQC spectra of Ile-^13^CH_3_-labeled Hsc70 (40 μM) in the absence (*black*) and in the presence of unlabeled SNN_P_ (*orange*) and SNC_P_ (*green*) peptides (200 μM).

To validate the Hsc70-binding observed in peptide arrays, we synthesized peptides corresponding to the sequences of SNAP-25 that showed strong binding in the SNN, LOOP, and LOOP-SNC domains, and named the corresponding peptides SNN_P_, LOOP_P_, and SNC_P_, respectively as shown in Figure 4*C*. Hsp70s consist of an nucleotide-binding domain (NBD) and a substrate-binding domain (SBD). Hsc70 works using an allosteric mechanism whereby substrate binding at the SBD stimulates the ATP hydrolysis rate at the nucleotide-binding domain. The basal ATPase rate of Hsp70 is low, usually about 0.05 ± 0.009 mol Pi/mol protein, and increases when it is stimulated by binding of a peptide to the canonical site in the SBD. We measured the ability of SNAP-25 peptides SNN_P_ and SNC_P_ to stimulate the ATPase rate of Hsc70 (Fig. 5*A*). SNN_P_ and SNC_P_ binding cause a 1.5-fold increase of the Hsc70 ATPase rate over basal (observed rates upon addition of SNN_P_ and SNC_P_ were 0.089 ± 0.008 and 0.080 ± 0.005, respectively). In order to confirm that peptides are indeed binding at the canonical binding site of Hsc70, we used the V438F mutant that blocks the binding site (37). Addition of SNN_P_ and SNC_P_ to V438F Hsc70 did not enhance its ATPase activity, supporting the conclusion that these peptides bind to Hsc70 at its canonical binding site (Fig. S3). The addition of CSPαJ stimulates the ATPase activity of Hsc70 ∼4-fold with and without peptides. However, the ATPase activity of Hsc70 was not further increased in the presence of nucleotide exchange factor, Apg2 (Fig. S2*C*). These results suggest that CSPα works in collaboration with Hsc70 by increasing its ATPase activity but does not affect the binding specificity of Hsc70 (Figs. 4*A* and 5*A*).

To further characterize the binding of the SNAP-25 peptides to Hsc70 we used competition assays as above. Peptides SNN_P_, LOOP_P_, and SNC_P_ were titrated against the protein-peptide complex of Hsc70 and FITC-p5 and fluorescence anisotropy was monitored to assess how well the SNAP-25 peptides displaced the model peptide. The results show that SNAP-25 peptides SNN_P_ and SNC_P_ bind to Hsc70 with significantly higher affinity than LOOP_P_ (Fig. 5*B*). This result together with the likely sequestration of the LOOP region when SNAP-25 is membrane-anchored led us to proceed in our studies using only SNN_P_ and SNC_P_ as representing mechanistically significant Hsc70 binding sites on SNAP-25.

To directly observe binding of SNAP-25 peptides to Hsc70, we used methyl-TROSY NMR. We recorded the spectra of complexes between peptides containing the Hsc70 binding sites of SNAP-25 and Hsc70 selectively labeled (^1^H,^13^ C) at Ile δ1-methyl positions in a ^2^H, ^12^C background. We had previously found using the *Escherichia coli* Hsp70, DnaK, that the δ1-methyl chemical shifts of Ile 401 and 438, which contact the central residue of the bound peptide (anchor residue, Fig. 5*C*), change markedly depending on the identity of the bound anchor residue and the orientation of the peptide backbone with respect to DnaK (38). In Hsc70, the side chains of Ile 403 and 440 are similarly arranged with respect to the anchor residue of the bound peptide as Ile 401 and 438 are in DnaK (Fig. 5*C*) allowing us to use their δ1-methyl chemical shifts to assess directly how the chaperone binds to SNAP-25 peptides SNN_P_ and SNC_P_. We confirmed the assignment of Ile 403 and Ile 440 chemical shifts by mutating these residues to Leu and collecting methyl-TROSY NMR spectra. The Hsp70 SBD site that binds the anchor residue (termed the 0^th^ pocket) is most frequently occupied by Leu or Ile and less commonly by Val, Pro, or Met (38, 39, 40, 41).

The signals for Ile 403 and 440 in the Ile methyl-TROSY of δ1-methyl labeled-Hsc70 indeed shift upon complex formation with SNN_P_ and SNC_P_, providing confirmation that these peptides bind at the canonical binding site of Hsc70. Binding of SNN_P_ (RRMLQLVEESK) causes the Ile 440 and 403 δ1-methyl chemical shifts to split into two resonances each, indicating that SNN_P_ binds in two modes: either using two different anchor residues (such as Leu 4, Leu 6, Val 7, or Met 3) or by binding the peptide in two alternative backbone orientations (N- to C- or C- to N- (38)).

The chemical shifts of Ile 440 also split into two signals upon binding of SNN_C_ (EREQMAISGGFIRR), consistent again with the population of two binding modes: either with two different anchor residues (Ile 7, Ile 12, or Met 5) or in opposite backbone orientations. The resonances for Ile 403 are typically less intense than Ile 440, therefore some split peaks are not observed. Studies are in progress to determine the precise binding mode.

### Hsc70 facilitates incorporation of SNAP-25 into the SNARE complex

**Figure 6:**
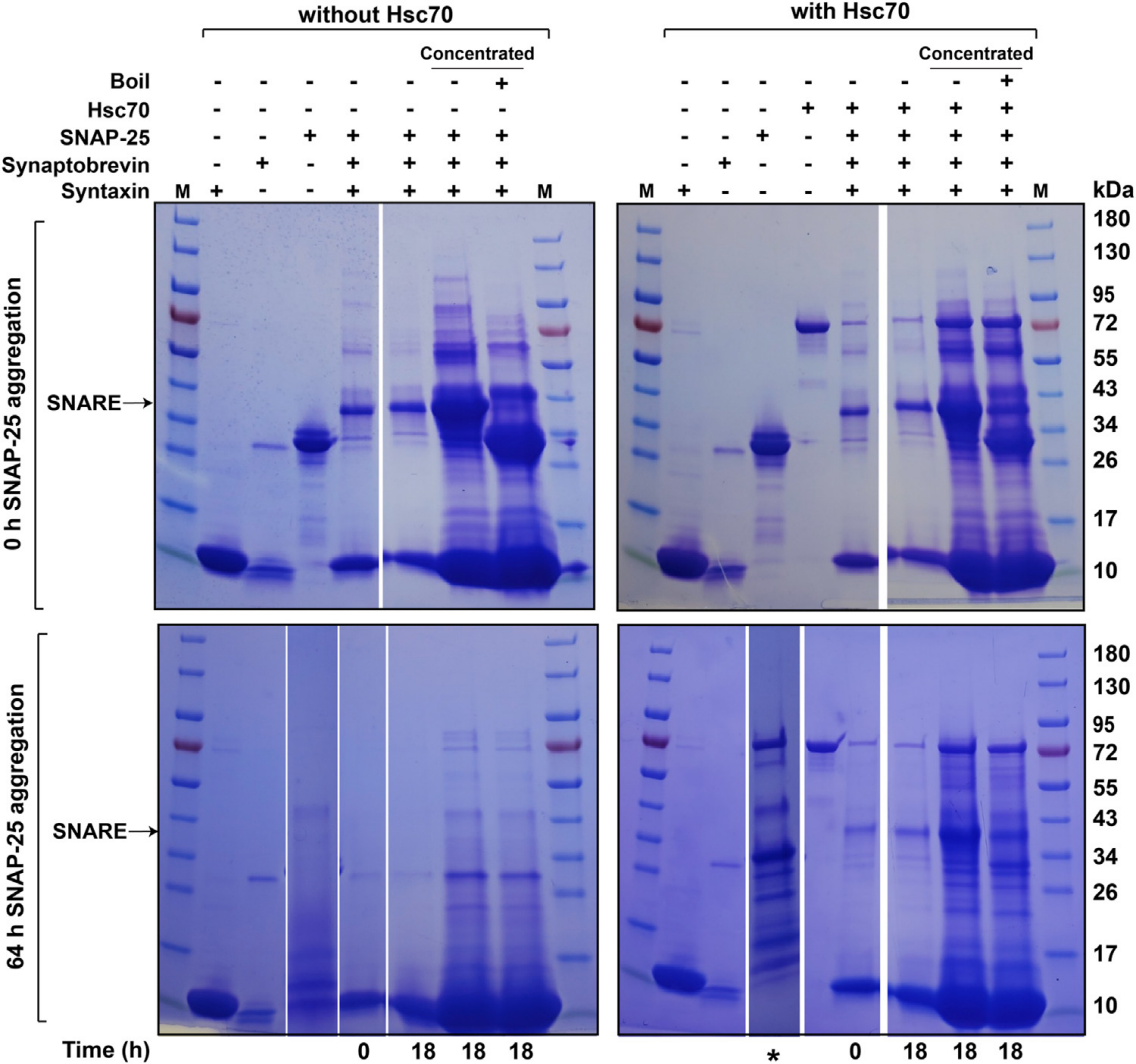
**Hsc70 facilitates incorporation of SNAP-25 into the SNARE complex *in vitro*.** SDS-PAGE of SNAP-25 taken from the aggregation reactions (Fig. 2) at 0 h and 64 h after the initiation, without (*left*) and with Hsc70 (*right*), and mixed with syntaxin and synaptobrevin for the time indicated below each SDS-PAGE. Uncropped gel images are provided in Figs. S4 and S5. SNAP-25 is expressed and purified from *E. coli*; a minor amount of the bacterial Hsp70 homolog DnaK (∼70 kDa) may copurify with SNAP-25, explaining the presence of higher molecular weight bands, with its proteolysis potentially occurring during ∼64-h SNAP-25 aggregation reactions. For synaptobrevin, we attribute the higher molecular weight bands to incomplete cleavage of GST-tagged synaptobrevin.

Our results indicate that the Hsc70 system disfavors the aggregation of SNAP-25 by binding to the SNN and SNC sites. We next show that while preventing aggregation the chaperone maintains a state of SNAP-25 competent to form the SNARE complex. We took aliquots of SNAP-25 at different time points of the aggregation reaction (at 0 h, in lag phase and at 64 h in the log or saturation phase, Fig. 2*A*) and mixed them with the SNARE motifs of syntaxin and synaptobrevin to allow the formation of the SNARE complex. Figure 6 shows that SNAP-25 taken at 0 h during the aggregation time course forms SDS-resistant SNARE complexes with synaptobrevin and syntaxin. However, SNAP-25 taken at 64 h after the aggregation reaction was initiated is unable to form the SNARE complexes. By contrast, in the presence of Hsc70, SNAP-25 removed from the aggregation reaction at both 0 h and 64 h can productively form a SNARE complex with syntaxin and synaptobrevin.

## Discussion

The SNARE complex formed by syntaxin, synaptobrevin, and SNAP-25 at the presynaptic terminal of a neuron is a mechanistically essential molecular machine involved in membrane fusion during neurotransmitter release (1, 42). Chaperones facilitating formation of the SNARE complex could be of prime importance to maintain the precise temporal and spatial fusion of synaptic vesicles with the plasma membrane. In this study, we propose that the Hsc70 chaperone keeps SNAP-25 in a folding-competent state before SNARE complex formation, and we thus used biochemical and biophysical techniques to explore the interaction of Hsc70 with SNAP-25. Our data provide a detailed picture of the mode of interaction of the Hsc70 chaperone system with SNAP-25.

Excitingly, this study of the Hsc70-SNAP-25 complex offers a structural explanation for the upstream events preceding SNARE complex formation. From a clinical perspective, point mutations within SNAP-25 result in disruption in the formation of the SNARE complex (43). Previous studies found that genetic polymorphisms of SNAP-25 are correlated with the progression of AD and PD; in addition, elevated levels of SNAP-25 proteins are exhibited in schizophrenia patients and bipolar disorders (12, 13, 44). These previous studies suggest the importance of maintaining SNAP-25 in the folding-competent state, as its concentration modulation and point mutations could directly impact SNARE complex formation. Therefore, our data lead us to hypothesize that the upstream functioning of Hsc70 as a SNAP-25 chaperone in SNARE complex formation plays a pivotal role in neurotransmitter release.

Previous studies have provided evidence for the aggregation properties of SNAP-25, and these authors also examined the SNAP-25-Hsc70 interaction by SDS-PAGE and pull-down assays (22). Our aggregation kinetics data revealed that Hsc70 effectively delays SNAP-25 aggregation, and the presence of the co-chaperone CSPα enhances the aggregation inhibition effect of Hsc70 (Fig. 2). This cooperative action of Hsc70 and its co-chaperone underscores the complexity of the chaperone system in preventing protein aggregation in neurons. Our results suggest that the CSPαJ domain assists Hsc70 by enhancing its ATPase activity (Fig. 6). Additionally, as CSPα is localized at the synaptic vesicle membrane and SNAP-25 is anchored to the plasma membrane, the association of CSPα-Hsc70-SNAP-25 would bring the two membranes into proximity and thus favor SNARE complex formation and membrane fusion. It has been known that Hsc70 can function as a foldase and as a holdase (45). Our results indicate that Hsc70 acts as a holdase for SNAP-25, releasing it to enable the formation of a regulatory SNARE complex crucial for vesicle fusion in neurons.

**Figure 7:**
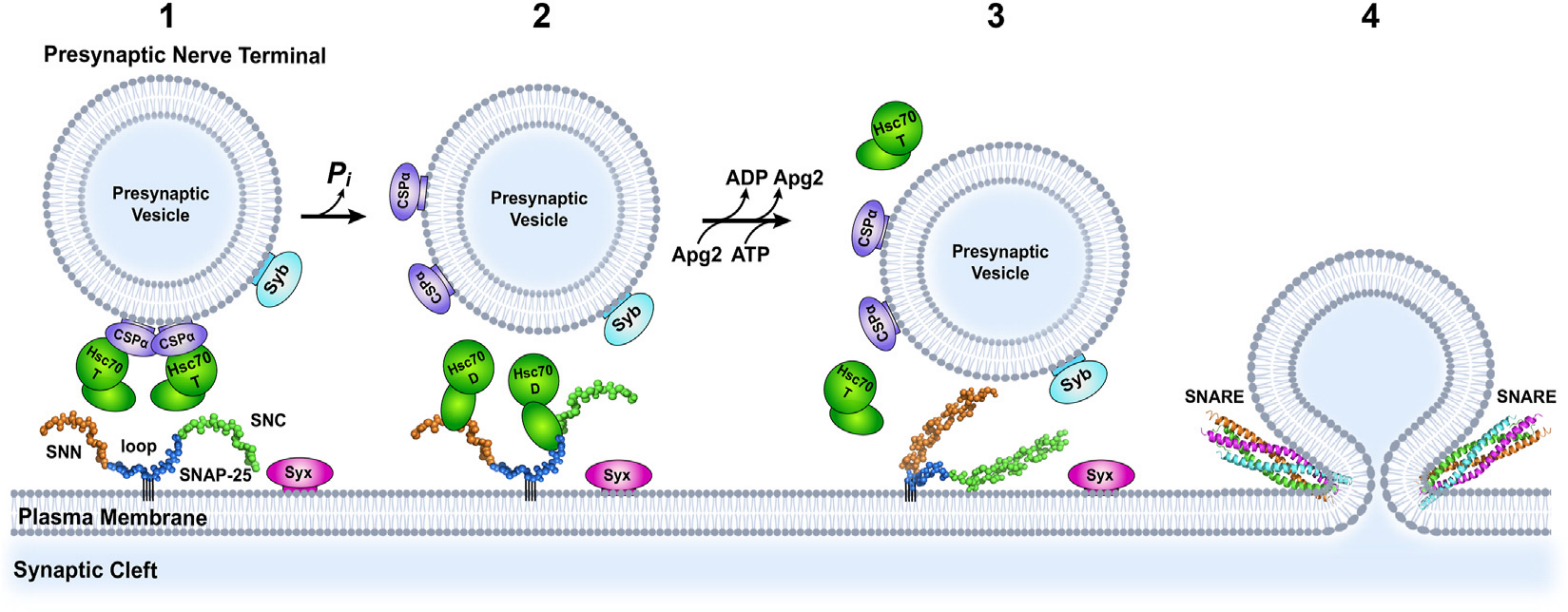
**A proposed model for Hsc70 chaperoning of SNAP-25.** SNAP-25 undergoes cycles of palmitoylation and depalmitoylation (55). For simplicity, we choose to show only the palmitoylated SNAP-25. Syntaxin and synaptobrevin are present on the plasma membrane and synaptic vesicle, respectively. In step 1, SNAP-25 is anchored on the plasma membrane, and ATP-bound Hsc70 is present in complex with the synaptic vesicle-resident CSPα. Step 2, Hsc70 binds to the N-terminal (SNN) and C-terminal (Loop-SNC) regions of SNAP-25, releasing CSPα and hydrolyzing ATP. Step 3, Upon ADP/ATP exchange facilitated by the nucleotide exchange factor, Apg2, Hsc70 releases SNAP-25 in a state competent for SNARE complex formation. In step 4, SNAP-25, syntaxin, and synaptobrevin join to form the SNARE complex.

Using biochemical assays, including pull-down assays, CD spectroscopy, peptide array screening, and NMR, we identified the binding sites of Hsc70 on SNAP-25 and elucidated the structural basis of their interaction. Using a peptide array, we identified three major and a small number of minor Hsc70 binding sites on SNAP-25 (Fig. 4*A*). Our binding competition assay demonstrated Hsc70 interacts with both the SNN and SNC fragments of SNAP-25, as well as the SNN and LOOP-SNC peptides, underscoring the multifaceted nature of the Hsc70-SNAP-25 interaction. The canonical binding site in the SBD of Hsc70 binds to SNAP-25 *via* SNN and a region spanning the C-terminal end of the LOOP and N-terminal of SNC domains. Interestingly, the N-termini of the SNARE components are proposed to assemble first in SNARE complex formation, followed by the C-termini of the SNARE components (46). Therefore, Hsc70 binding to the N-terminal regions of the SNN and SNC of SNAP-25 would be mechanistically advantageous *in vivo*. We propose a model (Fig. 7) in which the transient, regulated binding and release of Hsc70 to the N-terminal of SNAP-25 *via* the SNN and SNC regions prevent SNAP-25 aggregation and simultaneously facilitate active SNARE complex formation for vesicle fusion by making folding-competent SNAP25 available.

A recent report showed the MUN domain of the chaperone Munc13-1 binds to SNAP-25 in the loop region (47). However, as the loop region of SNAP-25 is palmitoylated and anchored to the plasma membrane in neurons, this interaction is unlikely to occur *in vivo* (1, 16). Alternatively, Munc13-1 and Hsc70 chaperones could work in collaboration to modulate SNAP-25 association with other SNARE proteins.

We took the advantage of the SDS-resistant nature of the SNARE complex (48) to demonstrate that while monomeric SNAP-25 is capable of forming SDS-resistant SNARE complexes with synaptobrevin and syntaxin, SNAP-25 taken during the log phase of the aggregation reaction loses this ability. This observation highlights the detrimental effect of SNAP-25 aggregation on SNARE complex formation, which is essential for neurotransmitter release. Importantly, our results revealed that the presence of Hsc70 during the aggregation process restored the ability of SNAP-25 to form SNARE complexes, suggesting that Hsc70 not only prevents SNAP-25 aggregation but also maintains it in a functional conformation, facilitating its participation in SNARE complex assembly. Our findings provide compelling evidence for the role of the Hsc70 chaperone system in preventing the aggregation of SNAP-25 and maintaining its competence to form the SNARE complex, thereby shedding light on crucial mechanisms underlying protein homeostasis in neuronal cells.

Overall, our findings contribute to a better understanding of the molecular mechanisms underlying obstacles to synaptic vesicle fusion in neurological disorders and provide potential therapeutic targets for intervention.

## Experimental procedures

### Protein expression and purification

Plasmids encoding the four proteins that form the SNARE complex (SNN_FR_ 11–82, SNC_FR_141–201, Syx 191–253, and Syb 29–93) were a generous gift from Professor Josep Rizo at the University of Texas, Southwestern, USA, and the plasmid encoding SNAP-25A was a gift from Professor Sreeganga Chandra at Yale School of Medicine, New Haven, USA. Using mutagenesis, the gene encoding SNAP-25A was changed to create the gene encoding SNAP-25B (the dominant form in neurons from adults) in the same plasmid. All four Cys residues of SNAP-25 were mutated to Ser and the Cys-less variant used for the experiments unless otherwise indicated. To investigate the role of the Hsp70 chaperone system in SNARE complex formation, we use the human constitutively expressed Hsp70, Hsc70, and the J-domain (residues 1–100) of human CSPα. The plasmid encoding the J-domain of human CSPα was kindly provided by Professors Lu-Yun Lian and Alan Morgan at the University of Liverpool, UK.

We expressed and purified Hsc70 (WT and the V438F mutant) using the protocol described in (49). Briefly, a plasmid encoding His-tagged Hsc70 was transformed into *E. coli* Rosetta (*DE3*) cells and plated on LB-agar plates supplemented with 100 μg/ml ampicillin and 100 μg/ml chloramphenicol. Bacteria containing the plasmid were grown in liquid LB supplemented with 100 μg/ml ampicillin and 100 μg/ml chloramphenicol at 37 °C. When the cultures reached an optical density at 600 nm of ∼0.5, proline (20 mM) and NaCl (300 mM) were added and the temperature changed to 30 °C. After 0.5 h, cells were supplemented with 0.6 M IPTG to induce protein overexpression and incubated for 6 h at 30 °C. Cells were then spun down at 4000 rpm for 20 min at 4 °C in a Beckman JLA 9.1 rotor, the cell pellet was resuspended in His-binding buffer (50 mM Tris, 10 mM imidazole, and 500 mM NaCl (pH 8.0)), the cell pellet was flash frozen in liquid nitrogen and stored at −80 °C.

For protein purification, the resuspended cell pellet was taken out of the freezer and 500 μl of Halt protease inhibitor cocktail (AEBSF, aprotinin, bestatin, E−64, leupeptin, and pepstatin A resuspended in 1 ml of water) was added. Cells were lysed by passage through a cell disruptor (Microfluidics), and the cell lysate was spun down for 45 min at 20,000 rpm at 4 °C in a Beckman JLA 25.5 rotor. The supernatant containing His-Hsc70 was loaded on a 15 ml nitrilotriacetic acid (Ni-NTA) agarose column attached to an AKTA Prime Plus FPLC System (Cytiva). After loading, the column was washed with His-Binding buffer, His-Washing buffer (50 mM Tris, 30 mM imidazole, 300 mM NaCl, (pH 8.0)), His-ATP buffer-no ATP (20 mM Tris, 5 mM MgCl_2_, 100 mM NaCl (pH 8.0)) and His-ATP buffer (20 mM Tris, 5 mM MgCl_2_, 100 mM NaCl, and 2 mM ATP) were flowed through the column to wash out any substrates bound to His-Hsc70. A gradient between 0 and 100% His-elution buffer (50 mM Tris, 300 mM imidazole, and 300 mM NaCl (pH 8.0)) was applied to the column to elute His-Hsc70. Eluted fractions were analyzed using 12% acrylamide SDS-PAGE and the tubes containing His-Hsc70 were pooled, supplemented with 1 mM DTT and His-Tobacco etch virus protease to remove the 6X His-tag, and incubated at 4 °C for 16 h. The sample was then buffer exchanged (either by dialysis or by ultrafiltration using Amicon Ultra Centrifugal Filter, 30 kDa molecular weight cutoff (Sigma-Millipore)) into His-binding buffer and loaded onto Ni-NTA agarose to separate digested product from the His-tagged proteins. Fractions containing Hsc70 were identified by SDS-PAGE, pooled, concentrated, and exchanged into HMK buffer (20 mM Hepes pH 7.4, 100 mM KCl, and 5 mM MgCl_2_). Aliquots were flash frozen in liquid nitrogen and stored at −80 °C.

All SNARE proteins and fragments were expressed and purified using the protocol described in (50). Briefly, plasmids encoding the His-tagged proteins (SNAP-25, Syx, SNN, and SNC) were transformed into *E. coli* BL21 (DE3) and plated on LB-agar plates supplemented with 100 μg/ml ampicillin. Liquid cultures of the bacteria containing plasmids were grown in LB at 37 °C with 100 μg/ml ampicillin until *A*_600_ reached ∼0.8. Protein overexpression was induced by addition of 0.4 M IPTG, and cultures were incubated for an additional 3 to 4 h at 37 °C. Cultures were spun down at 4000 rpm for 10 min at 4 °C in a Beckman JLA 9.1 rotor and resuspended in Buffer A (PBS 1X and 20 mM imidazole). The purification protocol is similar to WT Hsc70, except the buffers used were as follows: Buffer A (PBS 1X and 20 mM imidazole), Buffer B (PBS 1X, 20 mM imidazole and 1% Triton X), Buffer C (PBS 1X, 20 mM imidazole, and 1 M NaCl) and Buffer D (PBS 1X and 500 mM imidazole). After purification, fractions were collected and analyzed using 4 to 20% gradient SDS-PAGE. Fractions containing His-tagged protein were pooled, and the His-tag was cleaved by tobacco etch virus protease followed by Ni-NTA purification as described before. Pure fractions were pooled and stored with protease inhibitor at −80 °C. In the case of the synaptobrevin fragment, instead of Ni-NTA beads, GST beads were used, and the protein was eluted using PBS 1X with 10 mM glutathione as described in (50).

We expressed and purified CSPαJ tagged with both a SUMO and a His-Tag using the protocol described in (25). Briefly, plasmid encoding protein was transformed into *E. coli* BL21 (*DE3*) cells. Cells containing the plasmid were grown in LB-ampicillin at 37 °C overnight. A secondary culture was grown at 18 °C for 18 h, and protein expression was induced using 1 mM IPTG when *A*_600_ reached ∼0.6. Cells were harvested by centrifugation at 4000 rpm for 20 min at 4 °C in a Beckman JLA 9.1 rotor. Similar to Hsc70, CSPαJ was purified in the similar manner with different buffer. The composition of lysis buffer was 20 mM Tris (pH 7.5), 500 mM NaCl, 20 mM imidazole with Halt protease inhibitors; the wash buffer was 20 mM Tris (pH 7.5), 500 mM NaCl, 50 mM imidazole, and purified protein was eluted with a linear imidazole gradient from 50 mM to 500 mM. His-SUMO tags were removed using SUMO protease (Thermo Fisher Scientific, 1 unit per 1 μg protein), incubating the reaction overnight at 4 °C. Successfully cleaved CSPαJ was buffer exchanged into His-binding buffer by dialysis, and protein was flash frozen in liquid nitrogen and kept at −80 °C.

### CD experiments

The far-UV CD spectra (200–250 nm) were recorded on a Jasco J-1500 Circular Dichroism Spectrophotometer at 22 °C. The SNAP-25 WT, SNAP-25, and mixture of SNAP-25 and Hsc70 were taken in a 1 mm path length quartz cuvette. Figures show the average of ten scans where the buffer baselines were subtracted. Data were plotted using software using the Origin (Pro) program (OriginLab Corp).

### Native PAGE

Precast native PAGE gels (Bio-Rad, 4–20%) were used to separate proteins based on their conformation and charge. SNAP-25 (10 μM), Hsc70 (2 μM) and a mix of both samples were mixed with a nondenaturing loading buffer and directly loaded onto the gel without heating. Electrophoresis was carried out in Tris-glycine running buffer at 180V for approximately 1 h until the dye front reached the bottom of the gel. Gels were stained with Coomassie Brilliant Blue R-250 (Sigma-Aldrich) and destained in a methanol-acetic acid solution until clear bands were visible.

### Aggregation assay

Aggregation of SNAP-25 at 50 μM was followed by measuring the *A*_360_ in a 96-well plate using a BioTek plate reader (BioTek), at 37 °C with shaking at 300 rpm, in the absence and presence of Hsc70 and CSPαJ at 5 μM each, and Apg2 at 0.5 μM. The aggregation buffer included 20 mM phosphate buffer pH 7.4, 100 μM MgCl_2_, and 100 mM KCl, and an ATP-experiment was carried out using regeneration system (5 mM 2-phosphoenolpyruvate, 0.05 mg/ml pyruvate kinase, and 5 mM ATP), as described previously (51).

### Peptide array

A peptide array of SNAP-25 was obtained from CEM corporation. Peptides of 13-residue length, overlapping by 10 residues, and spanning the full sequence of SNAP-25 were immobilized on a nitrocellulose membrane using a PEG chemical linker. Before screening, the membrane was washed with 100% methanol and equilibrated with Tris buffered saline (TBS) 1X. The peptide array was incubated with Hsc70 (100 nM, 20 μM ADP and 200 nM ATP) in MP buffer (31 mM Tris–HCl (pH 7.6), 170 mM NaCl, 6.4 mM KCl, 0.05% Tween 20, and 5.0% sucrose) for 1 h at 25 °C while gently shaking. The array was washed with TBS-T (TBS plus 0.05% Tween 20) and probed with an anti-Hsc70 primary antibody (PA5-27337, Thermo Fisher Scientific) in 1X TBS-T for 12 h at 4 °C, washed three times with 1X TBS-T, and then incubated with an horse radish peroxidase-tagged secondary antibody. After three washes with 1X TBS-T, enhanced chemiluminescence (ECL) Western blot visualization solution was incubated on the membrane for 5 min. The chemiluminescence was captured in a G-box (Syngene), and the images were analyzed using ImageJ.

### Competition binding assay

Direct binding between the p5 peptide (QSRLLLSAPRRAA) and Hsc70 was carried out as described previously (52). Briefly, a concentration gradient between 0 and 100 μM Hsc70 protein was titrated against a constant concentration of 50 nM of FITC-labeled p5 (FITC-p5) in phosphate buffer (20 mM phosphate buffer, 100 μM MgCl_2_, 100 mM KCl, (pH 7.4)), 1 mM ADP, and 1 mM DTT. Reactions were incubated overnight at 4 °C in a 384 well plate. Fluorescence anisotropy was measured in a BioTek plate reader (BioTek) with excitation at 485 nm and emission at 516 nm.

The measured anisotropy values were converted to fraction bound (f_b_) using the equation:

$$fb=\left({r-r}_{0}\right)/\left(r_{b}{-r}_{0}\right)$$

where r is the anisotropy value at any point, r_0_ is the anisotropy value of FITC-p5 in the absence of Hsc70, and r_b_ is the anisotropy value of FITC-p5 when fully bound to Hsc70.

Using Origin (Pro) the curve of f_b_ *versus* concentration of Hsc70 was fitted to the equation:

$$fb=\frac{\left\{\left[P+S+K_{D}\right]-\sqrt{\left[{\left(xP+S+K_{D}\right)}^{2}-4*P*S\right]}\right\}}{2*S}$$

where *f*_*b*_ is fraction bound, *S* is the concentration of FITC-p5, *P* is the total concentration.

of added Hsc70, and *K*_D_ is the dissociation constant of the complex.

For competition assays, the concentrations of Hsc70 and FITC-P5 were kept constant at 5 μM and 50 nM, respectively, and the competitor substrate was titrated from 0 to 200 μM in phosphate buffer. Reactions were incubated overnight at 4 °C in 384-well plate. Fluorescent anisotropy was measured as described above. Each competition curve of *f*_*b*_ *versus* concentration of added peptide was fitted to the following equation to obtain the IC_50_:

$${fb=fb}_{0}+\left({fb}_{f}-fb0\right)*x/\left({x+IC}_{50}\right)$$

Using the Cheng and Prusoff Equation (53):

$$K_{i}{=IC}_{50}/\left[1+\left(\frac{R}{K_{D}}\right)\right]$$

IC_50_ was converted to *K*_D_ where *K*_*i*_ is the apparent dissociation constant of Hsc70 and the competitor peptide, *R* is the concentration of FITC-p5, and *K*_*D*_ corresponds to the affinity between FITC-p5 and Hsc70, as obtained with the direct binding assay.

### ATPase assay

We performed ATPase assays to observe the activity of Hsc70 in the presence of SNAP25 peptides. Hsc70 (2 μM) and peptide (200 μM) were incubated in HMK buffer with 1 mM DTT for 15 min followed by addition of 1 mM ATP and incubation at 37 °C for 1 h. Reactions were aliquoted into clear bottom 96 well plates in triplicates and diluted 4-fold in buffer (ATP concentration < 0.25 mM). A working solution from the Malachite Green Phosphatase Assay Kit (BioAssay System) was added to a final volume of 100 μl, and incubation continued for 15 min at 37 °C. Absorbance readings were taken at 620 nm using a BioTek plate reader. The baseline activity of CSPαJ and Apg2 proteins alone, which exhibited no ATPase activity, was subtracted.

### NMR

To prepare proteins with selectively labeled Ile, Leu, and Val methyl groups (*i.e.*, [U^2^H,^12^C,^15^ N]; Ileδ1-[^13^CH3]; Leu,Val-[^13^CH_3_, ^12^CD_3_]), cells were grown in D_2_O-based M9 minimal medium containing ^15^NH_4_Cl and ^2^H^12^C-glucose. The same protocol was used for bacterial growth and additionally, sodium salts of α-ketobutyric acid (Methyl^13^ C, 3,3-D2) and α-ketoisovaleric acid (3-Methyl^13^ C, 3,4,4,4-D_4_) acids were added (70 mg/L and 120 mg/L, respectively) 1 h before induction (54). All isotopically labeled compounds were obtained from Cambridge Isotope Laboratories, Inc.

NMR experiments were carried out at 25 °C on a 600 MHz Bruker Avance III spectrometer equipped with a CryoProbe. Data were processed with NMRPipe and analyzed using ccpNMR Analysis. For Methyl-TROSY experiments of the complexes between Hsc70 or with SNAP-25 peptides, the peptides were resuspended in H_2_O, adjusted to pH 7.4 and then lyophilized. Each lyophilized peptide was mixed with the protein in NMR buffer (10 mM potassium phosphate pD 7.4, 5 mM DTT, 0.02% sodium azide, 0.1 mM 4-(2-aminoethyl) benzenesulfonyl fluoride hydrochloride (AEBSF) in D_2_O) and incubated for 16 h at 4 °C. Concentrations used in NMR experiments were 40 μM ILV ^13^CH_3_ Hsc70 and 80 μM peptides.

### SNARE formation

The SNARE motifs were mixed in an equimolar ratio in the following order: synaptobrevin-2 (29–93), syntaxin-1A (191–253), and SNAP-25 taken from the aggregation reaction, in the presence of 1 M NaCl. The assembly reaction was incubated at 4 °C overnight while rotating. The SNARE motifs were concentrated using a 30-kDa molecular weight cutoff Amicon centrifugation filters and run on 4 to 20% gradient SDS-PAGE.

## Data availability

All data are included in this article or the Supporting Information.

## Supporting Information

This article contains supporting information.

## Conflict of interest

The authors declare that they have no conflicts of interest with the contents of this article.
