# Supplementary material for: The Hsc70 system maintains the synaptic SNARE protein SNAP-25 in an assembly-competent state and delays its aggregation[image]
Source: J Biol Chem. 2024 Nov 16;300(12):108001. doi: 10.1016/j.jbc.2024.108001 (PMC11697113; doi:10.1016/j.jbc.2024.108001)
Supplement: Supporting information [file mmc1.pdf]

SUPPORTING INFORMATION

The Hsc70 system maintains the synaptic SNARE protein SNAP-25 in an assembly-competent state and delays its aggregation

Karishma Bhasne[1], Antonia Bogoian-Mullen[1], Eugenia M. Clerico[1,*], Lila M. Gierasch[1,2,*]

[1]Department of Biochemistry & Molecular Biology and [2]Department of Chemistry

University of Massachusetts, Amherst MA, USA

*Address correspondence to Eugenia M. Clerico, eclerico@umass.edu; Lila M. Gierasch, gierasch@biochem.umass.edu

Contains: Supporting Figures SI 1 to SI 5

Figure SI 1. (A) Far-UV CD spectra of wild type SNAP-25 (black) and Cys to Ser mutant of SNAP-25 (red). (B) Far-UV CD spectra of SNAP-25 (black), Hsc70 (red), after mixing (green) and additive spectra of SNAP-25 and Hsc70 (blue).

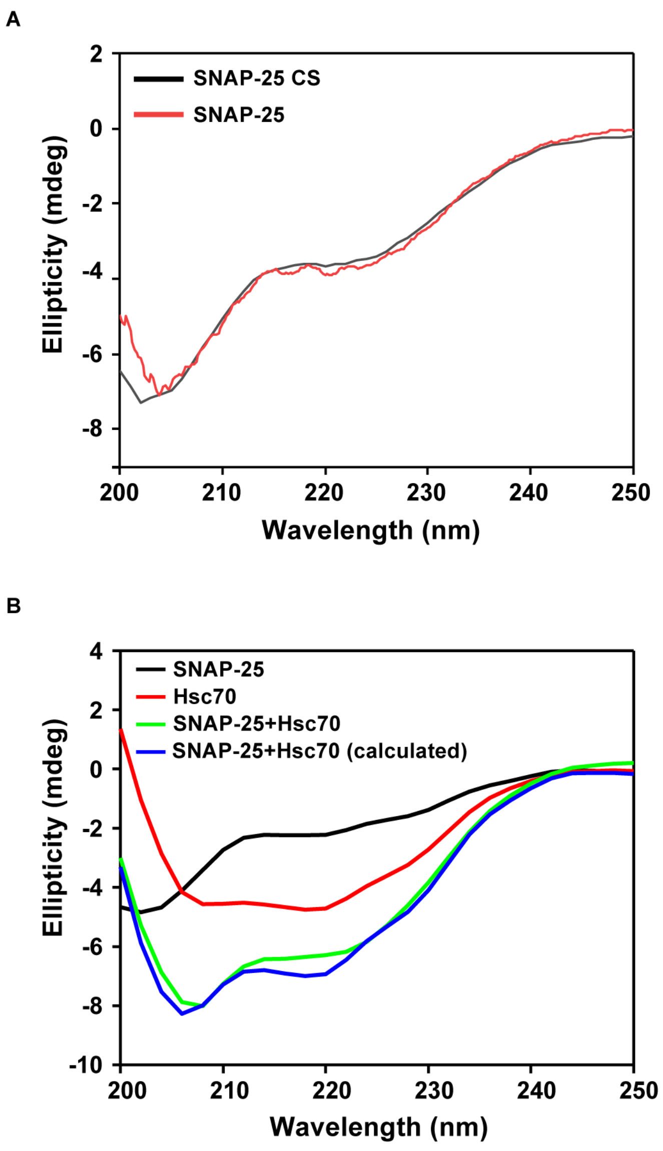

Figure SI 2. Apg2 enhances the effect of Hsc70 on SNAP-25. (A) Aggregation kinetics of SNAP-25 (50 µM) at 37 °C. OD_{600} of SNAP-25 was recorded in the absence (black) and in the presence of Hsc70, Hsc70+CSPα-J (blue) and Hsc70+CSPα-J+Apg2 (turquoise). Hsc70 (grey), CSPα-J (dark grey), and Apg2 (olive) alone exhibit no aggregation. The concentrations are SNAP-25 (50 µM), Hsc70 (5 µM), CSPα-J (5 µM) and Apg2 (0.5 µM). (B) Aggregation kinetics of SNAP-25 (50 µM) at 37 °C. OD_{600} of SNAP-25 was recorded in the absence (black) and in the presence of Apg2 (blue). Apg2 (blue) alone exhibit no aggregation. (C) ATPase activity of Hsc70 (at 2 µM) in the absence (grey) and presence of 200 µM SNN_P (orange) and L(Loop)-SNC_P (green) peptides, and in the absence and presence of CSPα-J and CSPα-J/Apg2. The statistical significance was calculated using one-way ANOVA test with Sidak's multiple comparison and the Hsc70 ATPase activity with peptides shows significant difference with Hsc70+CSPα-Peptides and Hsc70+CSPα-J+Apg2+Peptides. Error bars represent the SDs from three independent experiments.

SI Figure 2

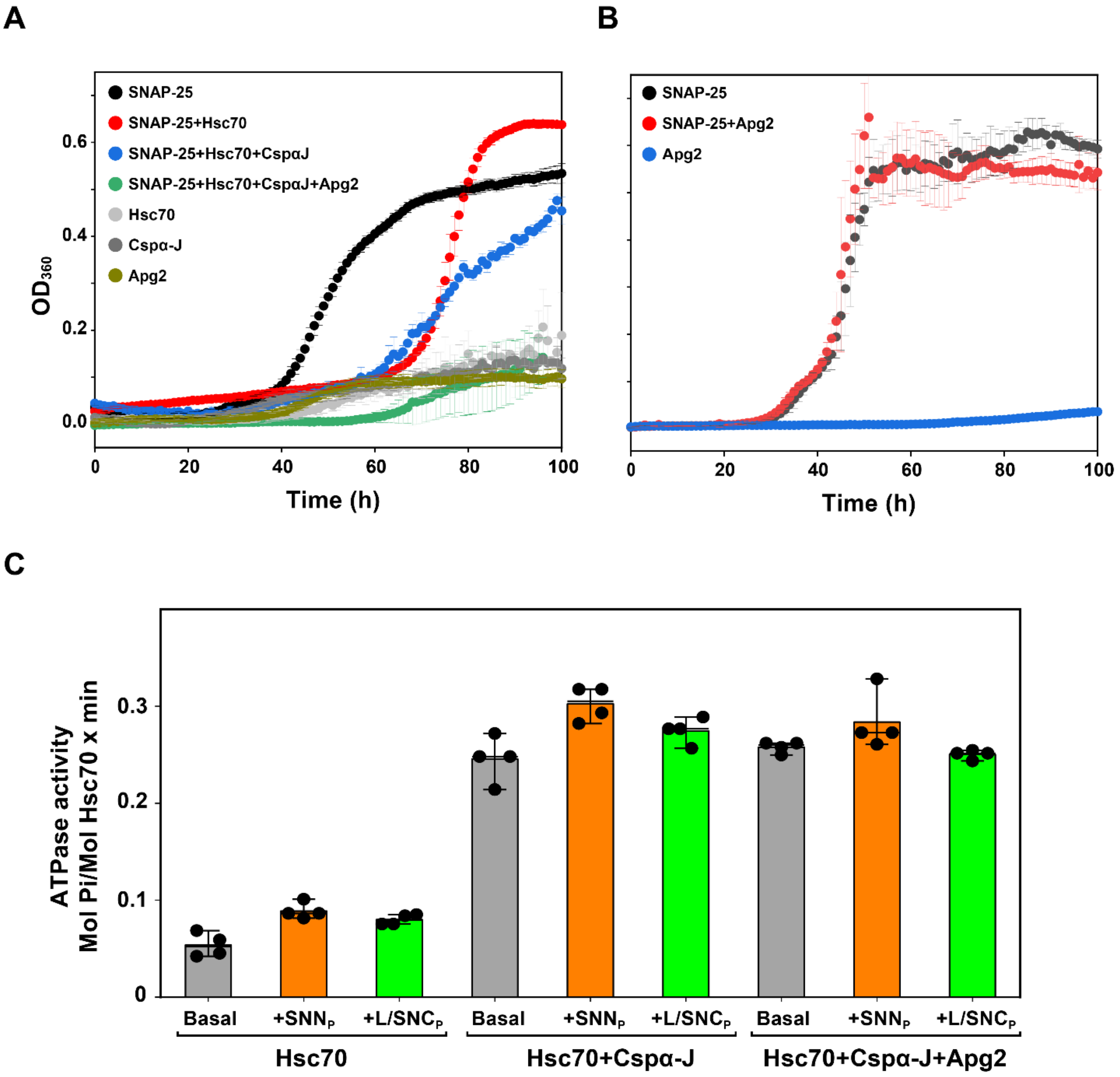
A
SNAP-25
SNAP-25+Hsc70
SNAP-25+Hsc70+CspαJ
SNAP-25+Hsc70+CspαJ+Apg2
Hsc70
Cspα-J
Apg2
OD360
0.0
0.2
0.4
0.6
0
20
40
60
80
100
Time (h)
B
SNAP-25
SNAP-25+Apg2
Apg2
0
20
40
60
80
100
Time (h)
C
ATPase activity
Mol Pi/Mol Hsc70 x min
0
0.1
0.2
0.3
Basal
+SNNP
+L/SNCP
Basal
+SNNP
+L/SNCP
Basal
+SNNP
+L/SNCP
Hsc70
Hsc70+Cspα-J
Hsc70+Cspα-J+Apg2

Figure SI 3. V438F mutation disrupts the Hsc70 canonical substrate binding site. ATPase activity of V438F Hsc70 (at 2 μM) in the absence (grey) and presence of 200 μM SNNP (orange) and L(Loop)-SNCP (green) peptides. Error bars represent the SDs from three independent experiments.

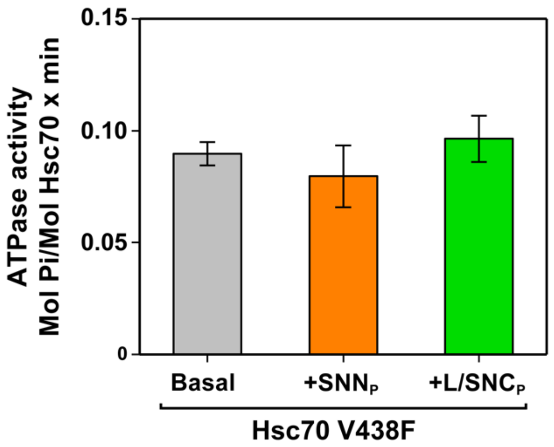

Figure SI 4. Uncropped SDS-PAGE images used in figure 6 (top panel). SDS-PAGE of SNAP-25 (left) and SNAP-25-Hsc70 (right) taken from the aggregation reactions (Figure 2) at 0 h and mixed with syntaxin and synaptobrevin for the time indicated below each SDS-PAGE. For synaptobrevin, we attribute the higher molecular weight bands to incomplete cleavage of GST-tagged synaptobrevin.

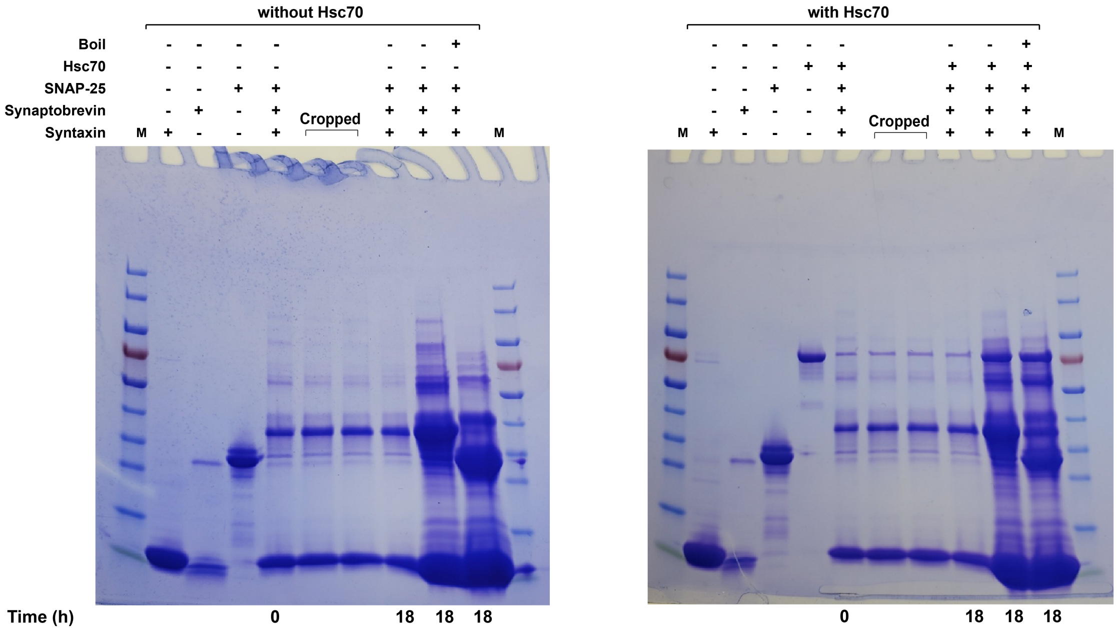

Figure SI 5. Uncropped SDS-PAGE images used in figure 6 (top panel). SDS-PAGE of (A) SNAP-25 and (B) SNAP-25-Hsc70 , and (C) aliquots of both reactions, taken from the aggregation reactions (Figure 2) at 64 h and mixed with syntaxin and synaptobrevin for the time indicated below each SDS-PAGE. SNAP-25 is expressed and purified from *E. coli*; a minor amount of the bacterial Hsp70 homolog DnaK (~70 kDa) may co-purify with SNAP-25, explaining the presence of higher molecular weight bands, with its proteolysis potentially occurring during ~64-hour SNAP-25 aggregation reactions. For synaptobrevin, we attribute the higher molecular weight bands to incomplete cleavage of GST-tagged synaptobrevin. * The SNAP-25 aliquot taken out from SNAP-25-Hsc70 aggregation at 64 h.

SI Figure 5

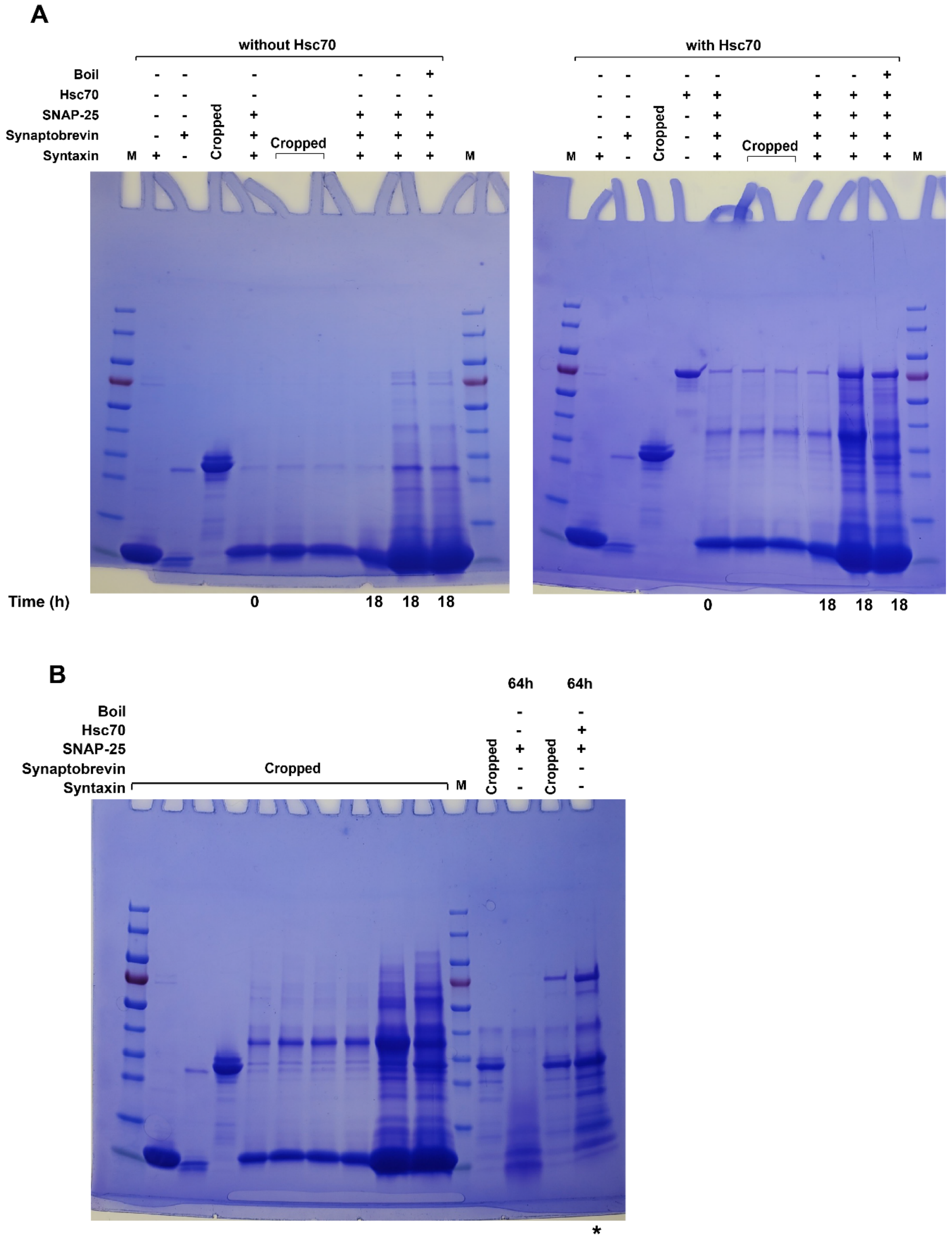
A
without Hsc70
with Hsc70
Boil
Hsc70
SNAP-25
Synaptobrevin
Syntaxin
M
Cropped
Time (h)
0
18
B
64h
Cropped
*
